# Malignant hypertension and acute aortic dissection associated with caffeine-based ephedra-free dietary supplements: a case report

**DOI:** 10.1186/1757-1626-2-6612

**Published:** 2009-04-03

**Authors:** Imdad Ahmed

**Affiliations:** 1 Department of Hospital Medicine, Regions Hospital, University of Minnesota Medical School, 640 Jackson Street, St. Paul-55101, USA

## Abstract

The use of weight loss dietary supplements is prevalent in the United States, and over the past decade, there has been tremendous growth of the use of these products. It is well documented that ephedra-based products are associated with various cardiovascular adverse effects. With new restrictions placed on such products, companies are now manufacturing caffeine-based ephedra-free herbal supplements. We present the case of 36-year old, previously healthy female who developed malignant hypertension and aortic dissection while taking various caffeine-based dietary supplements. Given the lack of research studies in regards to their safety and efficacy, judicious care should be taken with the use of dietary supplements, including those designated as ephedra-free.

## Introduction

The prevalence of obesity has increased markedly in the United States. Successful weight loss strategy involves long-term life style changes such as reducing calorie consumption and increasing physical activity [1]. In the United States, dietary supplements for weight loss are not recommended for losing weight due to concerns about efficacy and safety. Despite these rising concerns and several United States Food and drug Administration (FDA) warnings, supplements are an appealing alternative or adjunct for weight management for many people. Dietary supplements are widely available and there is little information available on who is using these products and how they are typically used [2]. It is well documented that ephedra-based supplements are associated with adverse reactions, especially cardiovascular and neurological injuries [3]-[5]. With new restrictions from FDA on ephedra, companies are now manufacturing caffeine-based ephedra-free dietary supplements and recent reports suggest that they may have potential, serious side effects that are similar to that of ephedra-based products [6,7]. We report the case of a patient who developed malignant hypertension and aortic dissection associated with the excess use of multiple caffeine-based ephedra free weight loss dietary supplements.

## Case presentation

A 36-year-old, previously healthy Hispanic female with no significant past medical history and no prior history of hypertension presented to the emergency room with sharp retrosternal chest pain radiating to the back. She was 165 centimeter tall and weighed 70 kilogram. She was a housewife and mother of two living children. She smoked one pack of cigarettes a day for more than ten years. For 3 months prior to presentation, she had been taking 18-20 tablets of caffeine-based weight loss pills daily, which was higher than the recommended daily dose. Each tablet contains 60-100 mg of caffeine and she was taking 1080 mg to 2000 mg of caffeine daily. She denied the use of other medications, stimulants, herbal, alcohol and illicit drugs. She said that she had lost approximately 20-25 kilograms in last 3 months. No family history of hypertension, diabetes or coronary artery disease. She denied palpitation, chest pain, insomnia, headache, abdominal pain or increase in urination.

**Figure 1:**
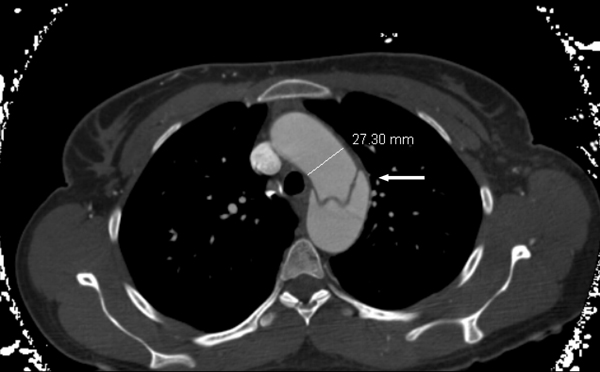
**Aortic dissection involving the thoracic aortic arch at the mid portion in the posterior aspect of the arch (thick arrow)**. Thoracic aorta was of normal caliber (27.3 mm).

**Figure 2:**
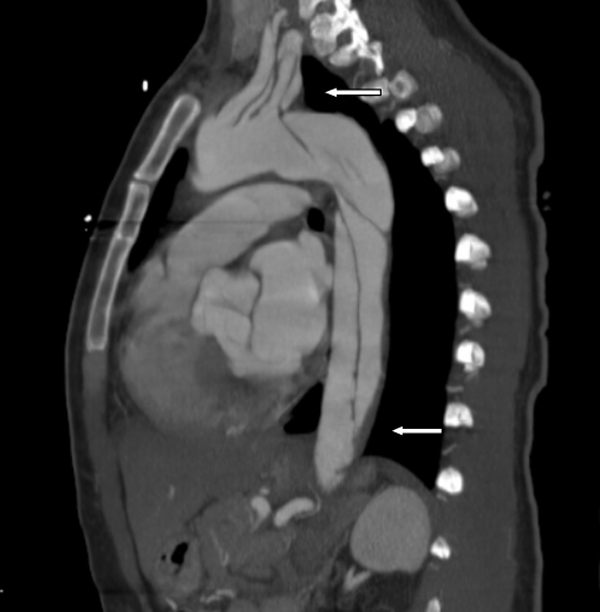
**Aortic dissection extending into the left subclavian and axillary arteries and to the level of bifurcation of the common iliac artery (thick arrows)**.

**Figure 3:**
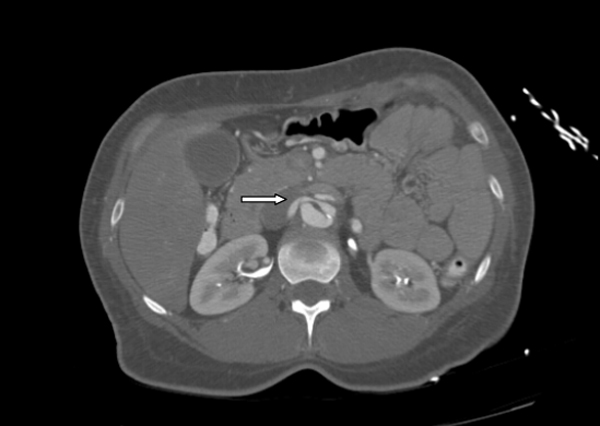
**There is narrowing of the origin of the right renal artery due to dissection (thick arrow)**.

On presentation, her blood pressure was found to be 220/110 mm Hg in right arm and 230/118 mm Hg in left arm. Her initial work-up included an electrocardiogram which showed T-wave inversion in leads III, aVF and V6. A complete blood count showed hemoglobin 12.9 g/dl (normal: 12.0-16.0 g/dl), white blood cell count 15.1 k/ul (normal: 4.0-11.0 k/ul) and platelet count 231 k/ul (normal: 150-450 k/ul). Serum basic metabolic panel showed sodium 132 mmol/L (normal: 135-145 mmol/L), potassium 3.3 mmol/L (normal: 3.5-5.3 mmol/L), chloride 103 mmol/L (normal: 95/105 mmol/L), bicarbonate 23 mmol/L (normal: 22-31 mmol/L), creatinine 1.3 mg/dl (normal: 0.6-1.3 mg/dl), calcium 8.3 mg/dl (normal: 8.6-10.3 mg/dl), and anion gap 6 mmol/L (normal: 7-17 mmol/L). Liver function test showed normal aspartate transaminase (15 U/L; normal: 0-55 U/L), alanine transaminase (23 U/L; normal: <45 U/L), alkaline phosphatase (55 U/L; normal: 34-104 U/L) and albumin (3.5 g/dl,normal:3.0-5.1 g/dl). Urinalysis showed only elevated specific gravity of 1.031(normal: 1.005-1.03). There were no crystals, protein and glucose in the urinalysis. Troponin I level was less than 0.030 ng/ml (normal: 0.0-0.049 ng/ml). Urine and serum toxicology screens were negative for cocaine, amphetamine, marijuana, benzodiazepines, barbiturates and phencyclidine. A chest X-ray did not show any infiltrate, consolidation, effusion or mediastinal widening. A computed tomography aortogram showed aortic dissection involving the posterior aspect of the arch extending into the common iliac arteries and right proximal external iliac artery. The dissection extended into the left subclavian and axillary arteries. There was narrowing of the origin of the right renal artery due to dissection (Stanford type B dissection) (Figure 1,Figure 2, & Figure 3.)

She was started on intravenous esmolol and nitroprusside for blood pressure control and was admitted to the medical intensive care unit. During hospitalization, her blood pressure remained well controlled with amlodipine. All other investigations for a secondary cause of hypertension were negative.Patient was discharged home and upon follow-up 2 week later, her blood pressure remained well controlled with low dose amlodipine.

## Discussion

To our knowledge, the patient described above represents the first reported case of aortic dissection due to malignant hypertension associated with caffeine-based weight loss dietary supplements. There has been tremendous growth in the use of weight loss dietary supplements over the past decade [8]. Coma, seizures, conduction abnormalities, hypertension, hepatotoxicity, and anti-cholinergic toxicity are previously reported medical complications attributable to inherent toxicity, adulteration, or inadvertent plant substitutions [9,11]. Such health problems can arise from improper use of the product; product content; product tampering; and product defects that can alter product quality, purity and composition [12]. A recent study showed that caffeine-based product significantly increased blood pressure by 9.6 mm Hg and heart rate by 16.7 beats per minute [13].

Xenadrine EFX-a caffeine-based supplement has been implicated in a case of exercise induced syncope associated with QT prolongation [6]. It is important to note that the caffeine-based product also contained a small amount of synephrine. This raises the possibility that caffeine may be working synergistically with synephrine to cause the elevations in blood pressure that were seen with Xenadrine EFX. [6]. Excessive use of caffeine-based dietary supplements, presence of host of other ingredients and their synergistic effects with caffeine, might explain the malignant hypertension in our patient. She was also a chronic smoker and it is quite possible that toxicity of high dose of caffeine was enhanced by chronic smoking.

## Conclusion

We have presented a case of potential life threatening complication from the use of dietary supplements marketed for weight loss. Literature review suggests that consumers of dietary supplements tend to be less likely to reveal their use of dietary supplements to their primary care giver than their use of medication [14]. Providing routine scientific based advice will be a challenge for health care professionals because of the increasing variety of nonprescription products on the market. It's important for the health care professionals to take an active role in educating patients in routine basis to make appropriate choices regarding the use of weight loss dietary supplements.

## List of abbreviations

FDA: Food and Drug Administration; g/dl: Gram per deciliter; k/ul: Thousand cells per microliter; mmol/L: Millmole per liter; mg/dl: Milligram per deciliter; U/L: Unit per liter; g/dl: Gram per deciliter; ng/ml: Nanogram per milliliter.

## Consent

Unfortunately, the patient could not be traced to obtain written informed consent. The patient however provided verbal consent during hospitalization for presentation and publication of the case in medical journals. I believe that this case report contains a worthwhile clinical lesson that could not be made as effectively in any other way. I expect that a reasonable person would not object to the publication since every effort has been made so that patient remains anonymous.

## Competing interests

The authors declare that they have no competing interests.

## Author's contribution

IA has reviewed all the information, did literature search and has drafted the manuscript. IA has read and approved the final manuscript.
